# Protective effect of G*roup B Streptococcus* type-III polysaccharide conjugates against maternal colonization, ascending infection and neonatal transmission in rodent models

**DOI:** 10.1038/s41598-018-20609-5

**Published:** 2018-02-07

**Authors:** Emiliano Chiarot, Angela Spagnuolo, Silvia Maccari, Eleonora Naimo, Alessandra Acquaviva, Raffaella Cecchi, Bruno Galletti, Monica Fabbrini, Elena Mori, Paolo Ruggiero, Guido Grandi, Maria Rita Fontana, Giuliano Bensi, Immaculada Margarit

**Affiliations:** 1 GSK, Siena, Italy; 2 Present Address: Hannover Medical School, institute of virology, Hannover, Germany; 3 Present Address: University of Trento, Centro di Biologia Integrata – CIBIO, Trento, Italy

## Abstract

Group B Streptococcus (GBS) is a normal inhabitant of recto-vaginal mucosae in up to 30% of healthy women. Colonization is a major risk factor for perinatal infection which can lead to severe complications such as stillbirth and neonatal invasive disease. Intra-partum antibiotic prophylaxis in colonized women is a safe and cost-effective preventive measure against early-onset disease in the first days of life, but has no effect on late-onset manifestations or on early maternal infection. Maternal immunization with capsular polysaccharide-based vaccines shows promise for the prevention of both early-onset and late-onset neonatal infections, although ability to prevent maternal colonization and ascending infection has been less studied. Here we investigated the effect of a GBS glycoconjugate vaccine since the very early stage of maternal GBS acquisition to neonatal outcome by rodent models of vaginal colonization and ascending infection. Immunization of female mice and rats with a type III glycoconjugate reduced vaginal colonization, infection of chorioamniotic/ placental membranes and bacterial transmission to fetuses and pups. Type III specific antibodies were detected in the blood and vagina of vaccinated mothers and their offspring. The obtained data support a potential preventive effect of GBS glycoconjugate vaccines during the different stages of pregnancy.

## Introduction

Group B streptococcus (GBS) colonizes the vagina and/or lower gastrointestinal tract of 10–30% of pregnant women and their neonates can become infected in utero or during delivery^1–3^. Neonatal GBS invasive disease can occur during the first week of life (early onset disease, EOD), or up to 3 months of age (late-onset disease, LOD) and main manifestations are bacteremia, sepsis, pneumonia and meningitis^4–6^. Besides asymptomatic cervico-vaginal/rectal colonization, GBS can cause a variety of maternal infections in the course of pregnancy and the postpartum period. These include urinary tract infections, bacteremia, sepsis, mastitis, vulvovaginitis, as well as chorio-amnionitis and endometritis^6,7^, that can result in severe maternal complications, fetal injury, pre-term delivery or stillbirth^8–10^.

Animal models of vaginal colonization have been developed with three main aims: investigating GBS factors involved in host interaction that may favor bacterial acquisition and persistence^11–14^, elucidating host innate immune responses^13,15^ and identifying potential preventive strategies^16–18^. By these models, it has been demonstrated that GBS carriage induces infiltration of lymphocytes and the production of soluble inflammatory mediators in the vaginal mucosa, and that GBS bacteria can persist in the genital tract for long periods by subverting innate immune defenses.

The effect of GBS ascending infection on adverse pregnancy outcome has been studied by bacterial intra-cervical inoculation in rabbits^19,20^, by catheterization/intra-amniotic instillation of GBS in non-human primates^21–24^ and by mouse intra-uterine, intra-peritoneal^25,26^ or intra-vaginal inoculation models^27–30^. Some of these studies have highlighted a key role for the GBS β-hemolytic pigment that can trigger neutrophil apoptosis and escape from neutrophil extracellular traps in the amniotic cavity^31^, breach of maternal-fetal barriers, macrophage pyroptosis and activation of the NLRP3 or the caspase 1 inflammasome^26^, suggesting that this virulence factor could be implicated in preterm birth and intra-uterine fetal injury. Of interest, evidence in primates has suggested that fetal injury can even occur despite clearance of choriodecidual infection by means of GBS-induced inflammatory mediators in the amniotic fluid that can enter in contact with the fetal lung or diffuse in fetal blood^22,24,32^.

Intra-partum antibiotic prophylaxis in GBS carrier women has led to a decrease in EOD incidence, but does not prevent early maternal infection and LOD. Concerns have also been raised regarding the emergence of antibiotic resistance^33–35^ and disruption of the neonatal microbiome^36^. The observed inverse relationship between maternal serotype-specific antibody levels against the GBS capsular polysaccharide (CPS) and the risk of neonatal invasive disease led to hypothesize that placentally transferred antibodies elicited by vaccination of pregnant women could protect infants from infection^37–39^. Based on the above and on pre-clinical evidence indicating protection of neonate mice from GBS lethal challenge by vaccination of dams^40^, maternal glycoconjugate vaccines based on the most common capsular polysaccharide serotypes are under development and have been shown to be safe and immunogenic in humans^41–43^.

The potential value of anti-capsular antibodies in preventing also GBS maternal colonization and the early stages of maternal ascending infection remains largely unexplored^44^. The aim of the present study was therefore to investigate whether maternal vaccination with capsular glycoconjugates could exert a protective effect during pregnancy, since the early stages of GBS maternal acquisition, to infection during gestation, vertical transmission to the fetus and post-delivery neonatal invasive disease. To this aim, we developed rodent models of GBS vaginal inoculation during pregnancy and assessed the effect of a vaccine composed of type III CPS conjugated to a detoxified Diphtheria Toxin (CRM_197_) in controlling maternal colonization, chorioamnion/placental infection, fetal transmission, neonatal outcome and colonization/infection rates in pups.

## Results

### Development of a mouse model of GBS perinatal infection

A mouse model was firstly developed to assess the effect of maternal vaccination with CPS conjugates on GBS vaginal colonization, ascending infection to uterus and neonatal outcome (mouse model of GBS perinatal infection, Supplementary Figure 1A). Female mice were inoculated with 10^8^ CFU of GBS COH1 (n = 28) or saline (n = 17) between 2 and 4 days before delivery. The COH1 serotype III strain selected for challenge belongs to the hypervirulent CC17 lineage that is frequently found in colonized women and is most commonly associated with neonatal invasive disease^45,46^.

**Figure 1:**
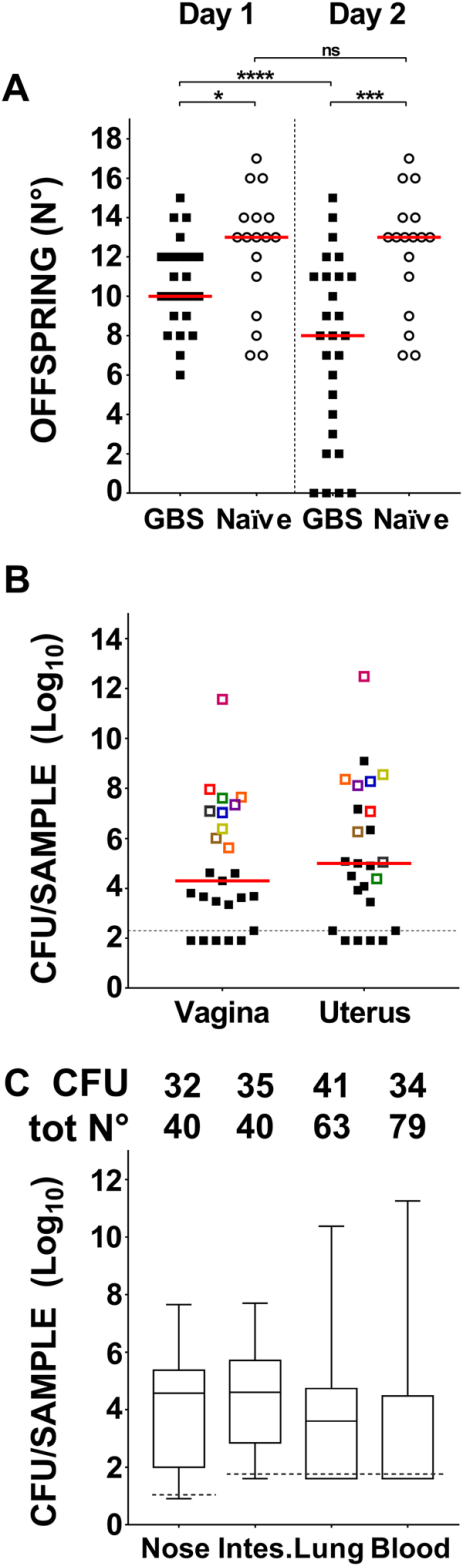
Mouse model of maternal vaginal colonization, ascending infection and transmission to offspring (perinatal infection). **(A)** Number of pups from mothers infected with GBS or placebo (Naïve) 1 day and 2 days after delivery. *P < 0.05; ***P < 0.001; ****P < 0.0001 (Mann-Whitney U-test to compare GBS infected group vs naïve group, Wilcoxon signed rank sum test to compare paired data at the 2 different time points). (**B)** Log_10_ CFU counts in whole vaginal and uterus homogenates obtained 2 days post-delivery from female mice infected with GBS. Animals showing vaginal CFU above 1.0 × 10^6^ are depicted in paired colors in the two columns. (**C)** Box-and-Whiskers blot of Log_10_ CFU/wash in nose, CFU/intestine (Intes.), CFU/lungs and CFU/ml in blood of offspring from GBS-infected mothers 2 days post-delivery. The number of samples where bacteria were found (CFU) and the total number of samples (tot N°) are reported above the graph. Median values and 10–25–75–90 percentiles are shown. The reported data derive from 4 independent experiments. In panels A and B, each dot represents one animal and the horizontal red line indicates the median value of the group. In panels B and C, the dotted grey line indicates the lower detectable value.

Live puppies were enumerated 1 and 2 days post-partum. As shown in Fig. 1A, the median number of pups on day 1 post-delivery in the GBS-infected group was lower than the median number of pups born to untreated animals (10 versus 13, P < 0.05). The following day there was a significant decrease in offspring survival in the infected group (69.4%, corresponding to a median of 8 live pups/dam) in comparison to the untreated group (99.5%, corresponding to 13 pups/dam) (P < 0.01); the number of surviving pups from GBS infected mothers presenting more than 10^8^ vaginal colony forming units (CFU/swab) was even lower (only 0–2).

The median numbers of bacterial CFUs in vaginal and uterus homogenates from GBS-inoculated mothers sacrificed 2 days post-delivery were 2 × 10^4^ and 1 × 10^5^ respectively (Fig. 1B). Animals showing high bacterial loads (above 10^6^ CFU) in the vaginal tissue generally presented high CFU counts in their uterus (see colored dots in Fig. 1B). No bacteria were found in the blood of any of the infected mothers (0/10) suggesting that bacterial spreading to uterus most likely occurred through an ascending path, although transient bacteraemia and haematogenous spread cannot be ruled out.

Finally, transmission of bacteria to neonates was investigated by measuring the number of CFU in nasal washes, the intestinal tract, the lungs and blood from 79 pups sacrificed on day 2 after birth. As shown in Fig. 1C, almost all pups presented bacteria in the nose (80%) and in the intestinal tract (88%), with median numbers as high as 6.05 × 10^4^ CFU/wash and 9.02 × 10^4^ CFU/sample respectively. Lung infection and bacteremia occurred less frequently (63% and 43% respectively) but with high bacterial loads (median values 3.7 × 10^4^ CFU/organ and 6.19 × 10^4^ CFU/ml of blood respectively). Overall, these data indicated GBS transmission from mother to offspring, resulting in neonatal mucosal colonization and, in some cases, systemic infection.

### Protective effect of maternal vaccination with type III  polysaccharide conjugates on mothers and neonates in the mouse model of GBS perinatal infection

**Figure 2:**
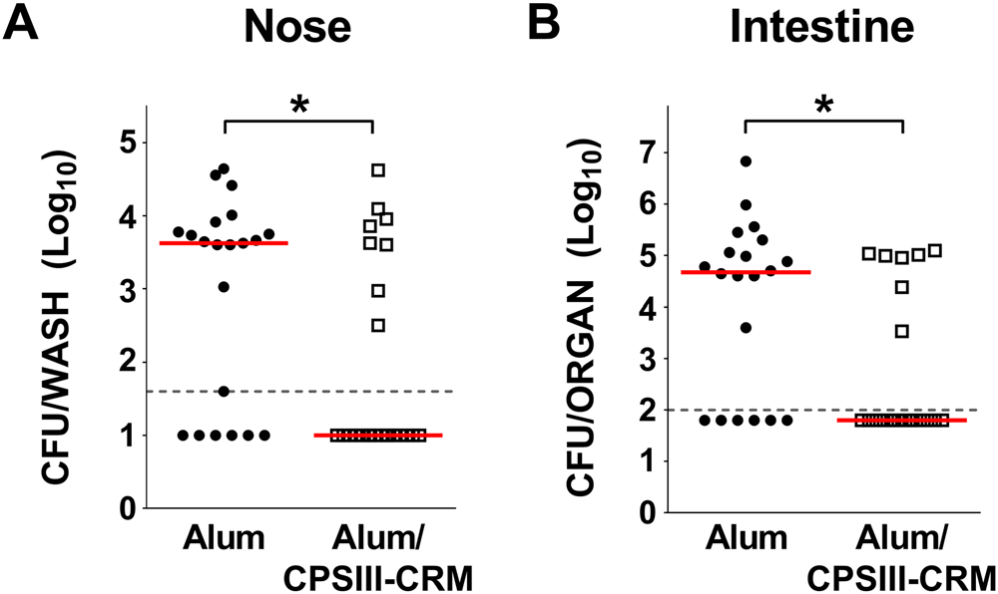
Effect of maternal vaccination with a type III glyconjugate on GBS transmission to pups. Log_10_ CFU in the offspring nose (CFU/wash) (**A**) and intestinal tract (CFU/organ) (**B**) from mice vaccinated with CPSIII-CRM or Alum alone and then infected with GBS III COH1. Data reported belong to 1 experiment, each dot represents one animal, the red horizontal line indicates the median value in each group and the dotted grey line the lower detectable value. *P < 0.05 (Mann-Whitney U-test).

The developed mouse model of perinatal GBS infection was then used to assess the effect of vaccination with a CPS conjugate on maternal infection and bacterial transmission to the offspring (see Supplementary Figure 1A). Female mice received 3 doses (days 0, 21, 35) of either type III CPS conjugated to genetically detoxified diphtheria toxin CRM_197_ (CPSIII-CRM) formulated in Alum (n = 8), or Alum alone as negative control (n = 7). Animals were mated on days 38–39 and intra-vaginally inoculated with 10^8^ CFU of GBS COH1 2–4 days before the expected date of delivery. Their pups were enumerated on days 1 and 2 post- delivery and then sacrificed for counting bacterial loads in their organs. In total, 120 pups were born from vaccinated dams and 105 from the negative control group. The following day, all the pups in the vaccine group were alive, while the offspring survival rate of the control group was 85% (P < 0.0001). Further, as shown in panels A and B of Fig. 2, there was a reduction in the frequency and the overall magnitude of GBS nasal and intestinal colonization in the vaccine group offspring (nose, 15/21 pups in the control group versus 8/24 in the vaccine group, P < 0.05 Fischer’s test; intestinal tract, 14/20 pups in the control group versus 7/21 in the vaccine group, P < 0.05, Fischer’s test). Spreading to lungs and blood in these experiments was limited in all groups and did not allow for a statistical comparison between the two groups.

### Protective effect of maternal vaccination on mothers and fetuses in a mouse model of GBS maternal ascending infection

**Figure 3:**
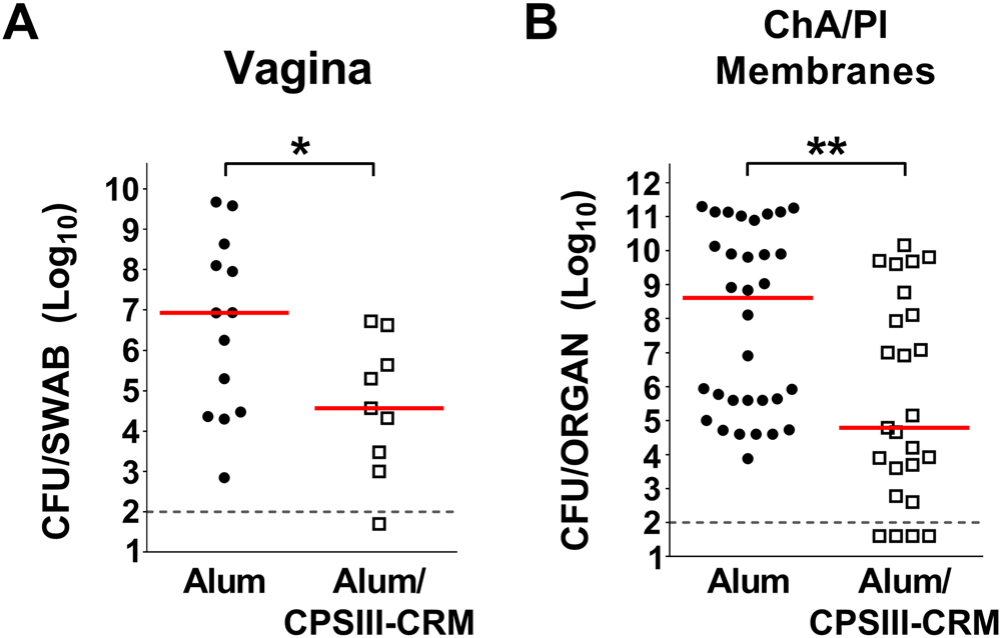
Effect of vaccination with GBS type III glycoconjugate on maternal ascending infection. Log_10_ CFU/organ in the vagina (**A**) and chorioamniotic/placental (ChA/Pl) membranes (**B**) of pregnant mice measured 2 days after intravaginal infection with GBS. Each dot represents one animal from 3 independent experiments, red horizontal lines indicate the median values and the grey dotted horizontal line the lower detectable value. *P < 0.05; **P < 0.01 (Mann-Whitney U-test).

The observed differences between the offspring of the vaccinated versus control mothers prompted more detailed investigation into the effect of vaccination on maternal colonization and ascending infection during the late stages of pregnancy. The mouse model was slightly modified so that pregnant vaccinated animals were sacrificed 2 days after infection (and therefore prior to delivery), to enumerate bacteria in maternal vaginal and chorion/placental membrane homogenates (mouse model of GBS ascending infection, Supplementary Figure 1B). As shown in Fig. 3A, a significantly lower bacterial load was found in the vagina of vaccinated dams as compared to sham immunized mice (2.4  log decrease in CFU median values, P < 0.05). Vaccination also showed a significant effect in reducing the number of bacteria at the level of chorioamniotic/placental tissues by almost 4 logs (Fig. 3B, P < 0.01).

Taken together, these data suggested that vaccination could reduce maternal vaginal colonization preventing GBS ascending infection and transmission to fetuses.

### Development of a rat model of GBS maternal ascending infection

To further investigate the vaccine effect on GBS transmission from the mother to the fetus at the placental level, we developed a rat model of colonization and ascending chorioamniotic infection that would permit monitoring bacterial spread at fetal level and histological analysis of fetal and maternal tissues (rat model of GBS ascending infection, Supplementary Figure 1C). Pregnant rats were intra-vaginally inoculated between 4 and 2 days before the expected delivery date with 10^8^ CFU of GBS COH1 and sacrificed 2 days later, prior to delivery. Bacteria were enumerated in the vagina and chorion/placental membrane homogenates, as well as in fetal lungs. Further, placental and fetal tissues were collected for histopathological analysis.

**Figure 4:**
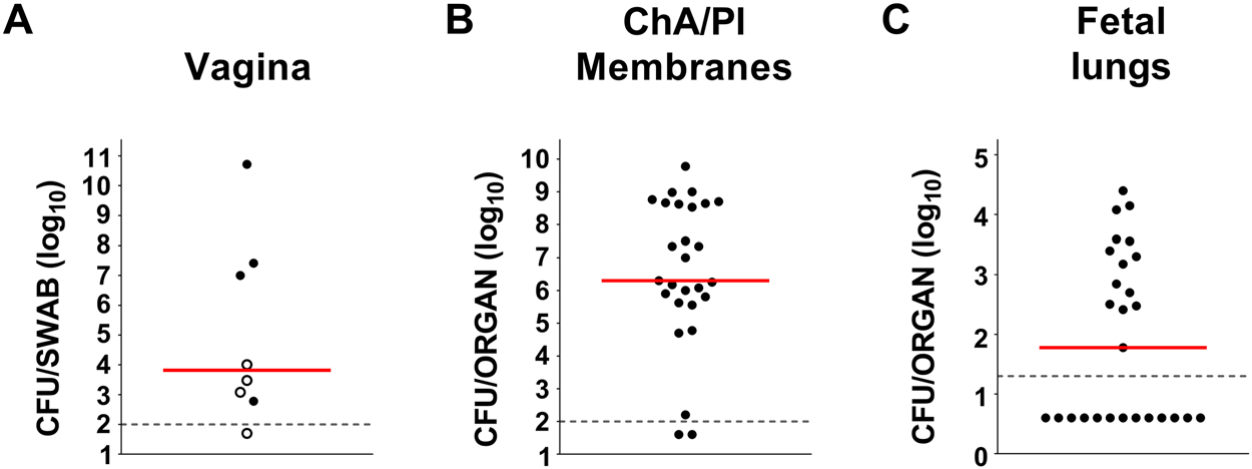
Rat model of GBS colonization, ascending chorioamniotic infection and fetal transmission. Log_10_ CFU counts in maternal vaginal swabs (CFU/swab) (**A**), chorioamniotic/placental (ChA/Pl) membranes (CFU/organ) (**B**) and fetal lungs (CFU/organ) (**C**) 2 days after intra-vaginal inoculation with GBS COH1. Each dot represents a single animal from 3 independent experiments and the red horizontal line indicates the median value of the group. The grey dotted horizontal line shows the lower detectable value. In A, full-black circles depict mothers presenting chorioamniotic/placental ascending infection. In B and C, only data relative to those mothers are shown.

Seven out of 8 infected animals presented GBS bacteria in their vagina (Fig. 4A) and ascending infection was detected in 4 of them (see closed circles in Fig. 4A and Fig. 4B). Bacteria were also found in the lungs of fetuses from mothers experiencing ascending infection (Fig. 4C).

**Figure 5:**
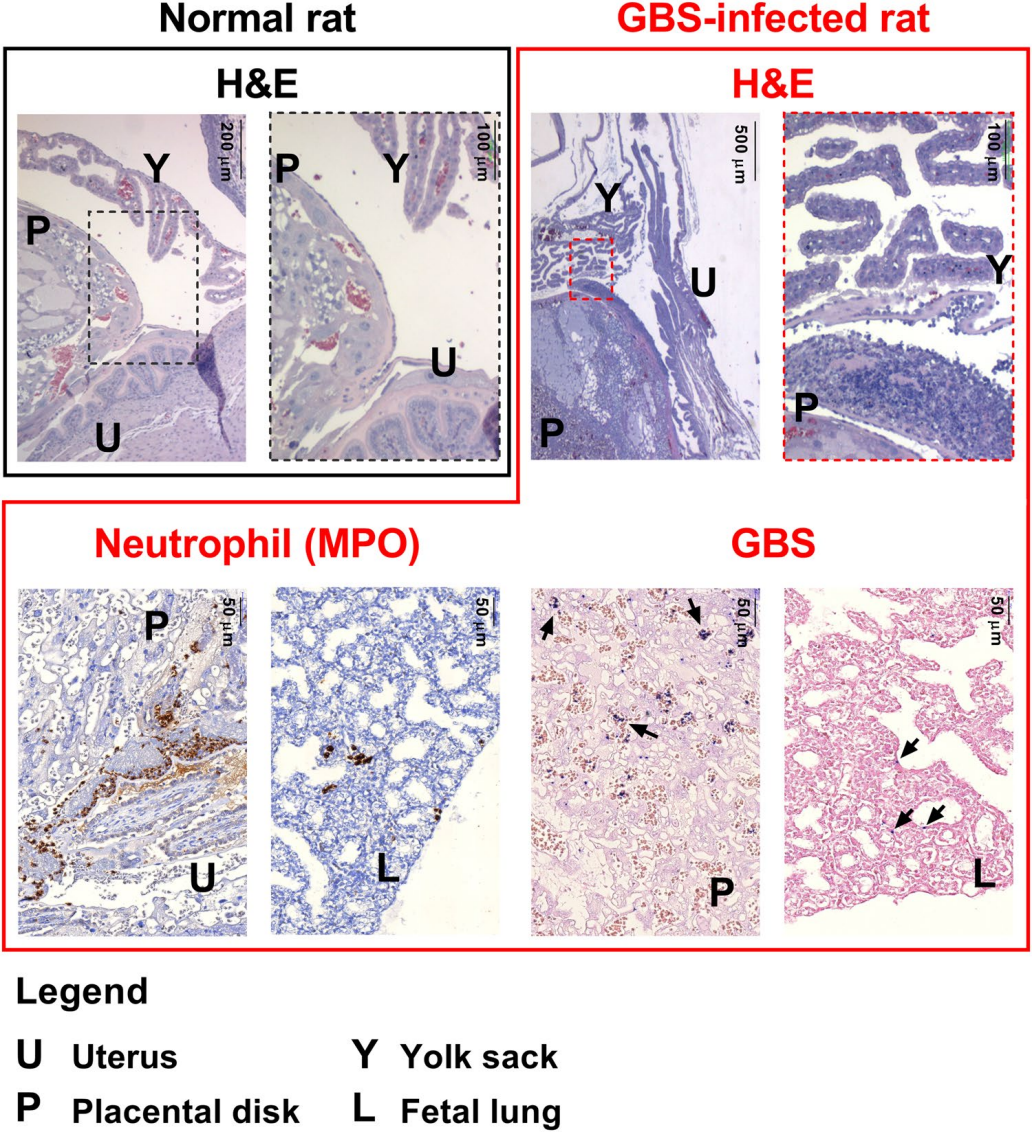
Histology and immunohistochemistry of rat colonization and ascending chorioamniotic infection. Histological samples of naïve rat fetuses (Normal rat) are surrounded by black boxes. Samples collected from infected animals (GBS-infected rat) are highlighted in red. In the upper part of the picture the histological analysis is shown, while immunohistochemistry for infected samples is reported at the bottom (Neutrophils and GBS). Black arrows indicate the presence of positively stained bacteria. No signals were observed neither in positive controls stained with isotypic controls nor in naïve animals stained for neutrophils and GBS. The scale for each single figure is reported in the upper half of the images. MPO = neutrophil myeloperoxidase.

Typical signs of inflammation compatible with chorioamnionitis were observed in tissues of infected rats when compared to naïve animals. Indeed, histopathological and immunohistochemical analysis revealed various degrees of inflammation severity associated with the presence of GBS (Fig. 5). Inflammatory cells, predominantly neutrophils and a lower number of mononuclear cells (prevalently lymphocytes), were observed within the chorioamniotic membranes (yolk sack and amnion), decidual membranes (uterus) and placental disks of infected animals. In most severely affected placental disks the Reichert’s membrane appeared disrupted by the infiltration of numerous inflammatory cells, prevalently neutrophils. Fragments of hyaline eosinophilic material (consistent with fragments of membrane) were seen scattered within the inflammatory exudate. Small areas of hemorrhagic and necrotic tissue, often containing aggregates of dark blue round bodies consistent with bacteria, were also detected within areas of inflammation.

### Protective effect of maternal vaccination with polysaccharide conjugates on mothers and fetuses in the rat model of ascending infection

We then used the developed rat model of ascending infection to assess whether a conjugate vaccine could provide maternal and fetal protection from a pre-delivery GBS intra-vaginal challenge. Female rats were vaccinated with Alum adjuvanted CPSIII-CRM on days 0, 21 and 31 prior to mating (days 38–39), and challenged with GBS 2 days prior to delivery (see Supplementary Figure 1C).

**Figure 6:**
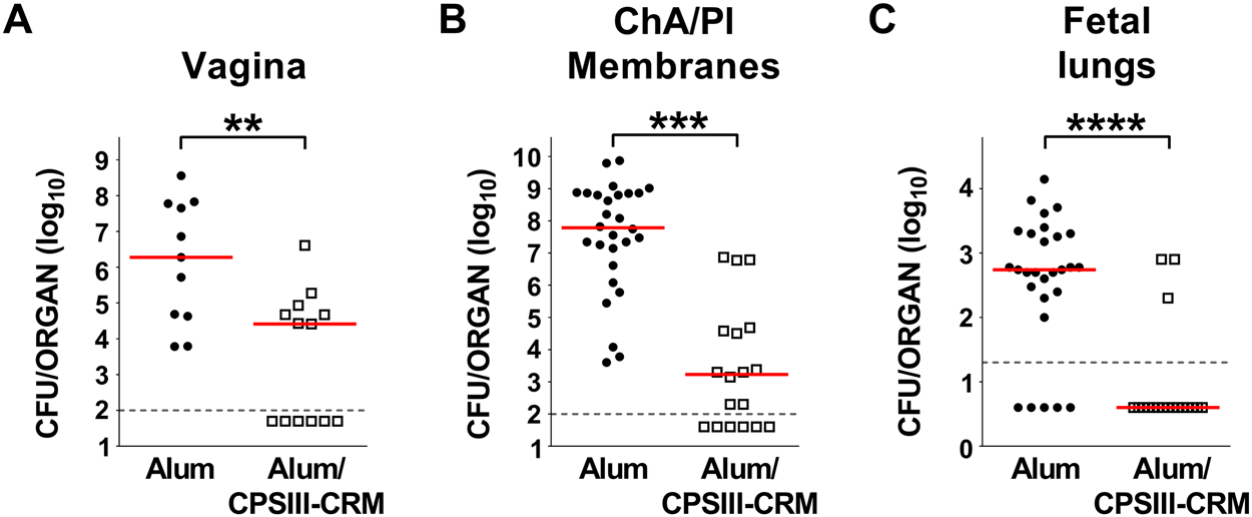
Effect of vaccination with CPSIII-CRM on rat maternal ascending infection and fetal transmission. Log_10_ CFU numbers of GBS COH1 in the vagina (CFU/swab) (**A**) and chorioamniotic/placental (ChA/Pl) membranes (CFU/organ) (**B**) of pregnant rats, and fetal lungs (CFU/organ) (**C**) measured 2 days after intra-vaginal infection. Samples were collected during 5 independent experiments. Each dot represents a single animal and the red horizontal line the median value of the group. The grey dotted horizontal line shows the lower detectable value. In (**B**) and (**C**) only data from animals experiencing ascending infection are shown. Samples from 6 (negative control) or 3 (vaccinated) mothers are reported. **P < 0.01; ***P < 0.001; ****P < 0.0001 (Mann-Whitney U-test).

As shown in Fig. 6A, immunization with CPSIII-CRM resulted in a significantly lower frequency of GBS colonized mothers (11/11 negative control group vs 7/13 in the vaccinated group; P < 0.05) and also in a significant decrease in the magnitude of carriage by more than 70-fold (from a median of 1.9 × 10^6^ CFU/sample to 2.6 × 10^4^ CFU/sample, P < 0.01). We also observed a trend towards a reduction in the frequency of ascending infection (3/13 of the vaccinated dams compared to 6/11 of the control group, P = 0.21) and significantly milder infection of chorioamniotic/placental membranes in vaccinated rats, with a 4.6 log reduction in the median number of CFU compared to control (Fig. 6B, P < 0.001). Finally, vaccination prevented GBS transmission to fetuses, with significantly lower numbers of bacteria recovered from their lungs (Fig. 6C, median CFU below the detection limit compared to 5.5 × 10^2^ in the control group, P < 0.0001).

**Figure 7:**
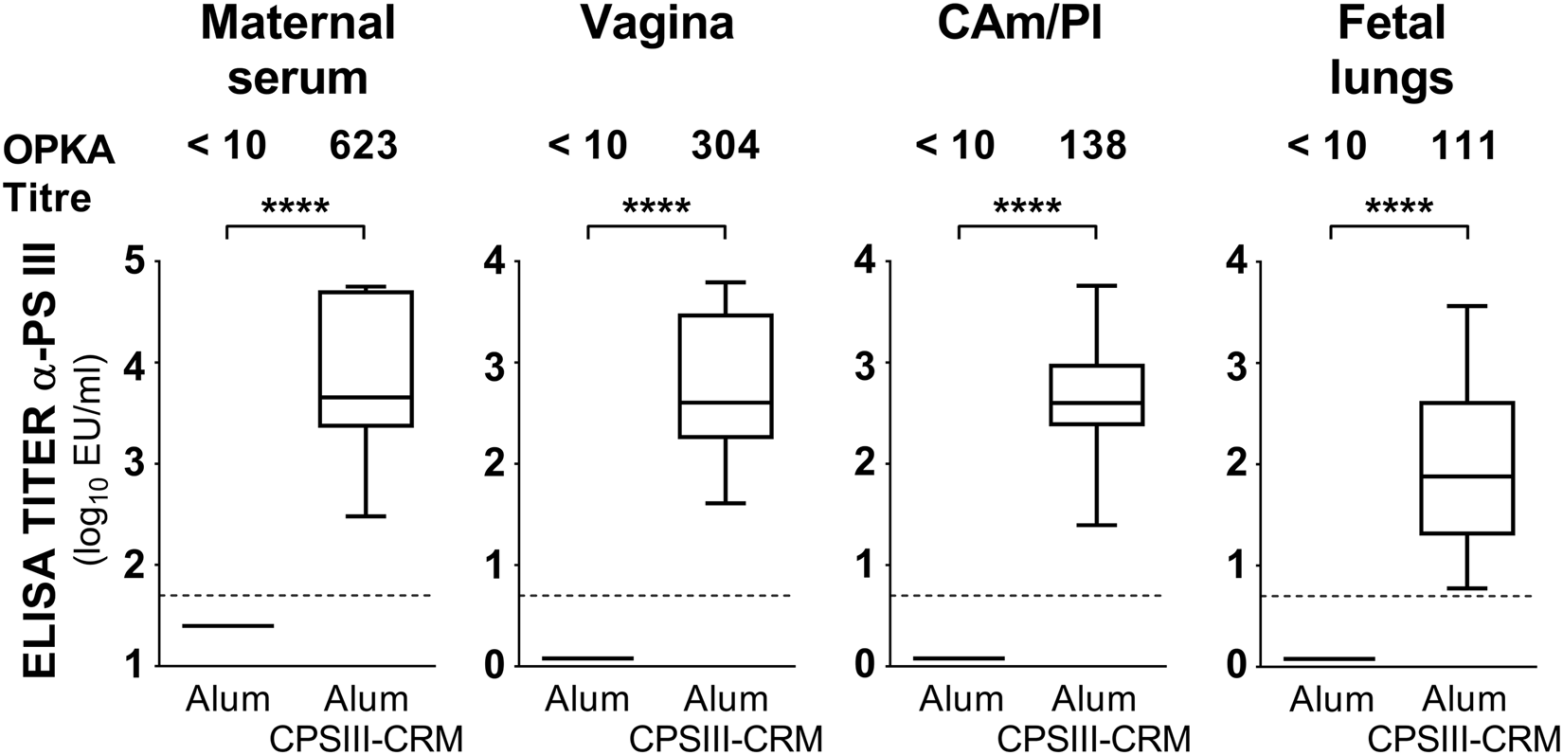
Anti-CPSIII IgG and OPKA titers in vaccinated female rats, and recruited at the sites of maternal and fetal infection. Log_10_ anti-CPSIII IgG titres measured in the indicated sites 2 days after intra-vaginal infection with GBS. The box and whiskers graphs show the median value of the group together with 10–25–75–90 percentiles. The numbers above indicate the median OPKA titres of single sera or pooled tissue homogenates. Presented data belong to 5 independent experiments. The grey dotted lines depict the lower detectable value. ****P < 0.0001 (Mann-Whitney U-test).

On the basis of the observed protective effect of the CPS glycoconjugate, we investigated anti-CPSIII IgG and functional antibody levels by ELISA and opsonophagocytic killing assay (OPKA), in maternal and fetal sites of immunized animals. As shown in Fig. 7, anti-type III IgG were found in maternal blood, vagina and chorioamniotic/placental membranes of immunized rats, as well as in fetal lungs. Further, antibody-mediated phagocytic killing was measured in samples collected from both maternal and fetal sites (Fig. 7).

To investigate the vaccine effect on inflammatory responses to GBS infection, we compared cytokine levels in the blood and tissue homogenates of vaccinated and control groups 2 days post-infection. The levels of some of the investigated cytokines were lower in vaccinated animals compared to control unvaccinated rats, with a significant decrease of IL-1β and MIP-3 in vagina and TNF-α and IL-10 in chorioamniotic/placental membranes (Table 1).

**Table 1:** CPSIII-CRM vaccinated rats present reduced signs of inflammation upon GBS infection.

Organ	Cytokine (ldv, pg/ml)	Treatment/ (Number of samples)	Mean (pg/organ)	Standard deviation (pg/organ)	P val (Two-tailed Mann-Whitney U-test)	Ratio (Mean Alum vs Alum/CPSIII-CRM)
Vagina	IL-1β (40)	Alum (11)	25097	39691	**0**.**018**	5.83
		Alum/ CPSIII-CRM (13)	4304	2186		
	MIP-3α (350)	Alum (11)	4202	5126	**0**.**024**	2.37
		Alum/ CPSIII-CRM (13)	1772	78		
Chorioamniotic/placental Membranes	IL-10 (660)	Alum (16)	2724	674	**0**.**002**	1.31
		Alum/ CPSIII-CRM (23)	2076	906		
	TNF-α (70)	Alum (16)	335	433	**0**.**023**	2.08
		Alum/ CPSIII-CRM (23)	161	102		

Legend: ldv = lower detectable value.

Data belong to 5 independent experiments.

For chorioamniotic/placental membranes samples, only materials from animals experiencing ascending infection were analyzed.

## Discussion

Maternal vaccination is considered the best preventive measure against Group B Streptococcus (GBS) invasive disease in the neonates^47–49^. Indeed, while IAP has been shown to be safe and effective against EOD, it is ineffective against LOD and has no impact on maternal recto-vaginal colonization or chorioamnionitis, the main risk factors for neonatal transmission^5,50–52^. Our results in rodent models suggest that immunization with capsular polysaccharide conjugates could be effective not only in reducing neonatal invasive disease, but also maternal GBS colonization and infection during pregnancy.

GBS is not a normal inhabitant of the rodent recto-vaginal tract, and rodents present only partial similarity to humans regarding hormonal cycling, vaginal pH and microbiome and placental/chorion characteristics. Despite this evidence, murine models of GBS vaginal colonization and ascending infection have recently provided important insights into virulence factors promoting maternal colonization/infection that may affect pregnancy^28,29^.

Here we show that rodent models can also represent a useful tool for predicting the effectiveness of preventive strategies against GBS infection during pregnancy. The effect of maternal vaccination on vaginal colonization, ascending infection and the offspring outcome was explored using a mouse model. Further, a maternal infection rat model allowed monitoring bacterial spread at the fetal level, histological analysis of fetal and maternal tissues, and evaluation of cytokines in the maternal blood and reproductive tract. The observed GBS ascending infection in both rodent models presented several features in common with human disease. Similar to humans, ascension of bacteria from the mouse and rat maternal vaginal mucosae resulted in intra-uterine infection with variable pregnancy outcome. In both models the risk of ascending infection was proportional to the density of colonization, and chorioamnionitis resulted in inflammatory disease, as also observed during human maternal infection^5,53^. Further, EOD seemed to evolve very rapidly as it usually happens in humans^4^. Bacteremia in the mouse pups was less frequent than mucosal colonization (40% vs above 80%) but when it occurred, the pups showed high bacterial loads and some of them died within 2 days post-delivery. Moreover, colonization of mouse dams during delivery resulted in mucosal colonization of the offspring, which is an important risk factor for LOD^54,55^. Remarkably, besides reducing maternal ascending infection and bacterial loads in fetal tissues, vaccination with the CPS conjugates also limited colonization of the puppies mucosae, suggesting a potentially additional effect of vaccination in the prevention of LOD.

In experiments conducted in rhesus monkeys, Larsen and colleagues observed an inverse association between the concentration of antibodies to type III GBS and neonatal susceptibility to intra-amniotic infection^56^. More recently, Baker and colleagues observed decreased GBS colonization persistence in wild-type non-pregnant mice that had undergone a previous colonization episode with the same strain and in those intra-nasally (but not intra-muscularly) immunized with whole killed bacteria prior to GBS vaginal inoculation^57^.

To our knowledge, the study presented here demonstrates for the first time that systemic immunization of female rodents with GBS conjugated polysaccharides elicits high levels of specific IgG that can limit new vaginal acquisition during pregnancy, ascending infection as well as the rate and degree of neonatal mucosal colonization and systemic infection. Evidence suggesting a role for IgG in controlling GBS vaginal colonization was recently obtained by Baker and colleagues, who reported that both B-cell deficient virgin mice unable to produce antibodies and mice lacking the neonatal Fc receptor (FcRn) mediating IgG transport to the vaginal mucosa exhibited prolonged colonization compared to wild type animals^57^.

Opsonophagocytosis has been recognized as the most important host defense mechanism to clear GBS infection^58^. Moreover, in a longitudinal sero-epidemiological study conducted in pregnant women, higher opsono-phagocytic killing (OPK) titers to GBS CPS types Ia and III were associated to a lower risk of recto-vaginal acquisition of strains belonging to the homologous serotype^44^. Here we show that anti-capsular antibodies from maternal blood, vagina and chorio-placental tissues and fetal lung homogenates of vaccinated rats were able to mediate opsonophagocytic killing *in vitro* (Fig. 7). Since the GBS capsular polysaccharide seems not to be involved in adherence to the vaginal mucosae^59^, the reduction in vaginal bacterial load observed after vaccination might depend on IgG-mediated killing of the bacteria by neutrophils present in vagina instead of inhibition of adherence to mucosal tissues.

Inflammation strongly contributes to worsening the final outcome of chorioamnionitis^60^ and we observed that vaccinated animals presented lower concentrations of pro-inflammatory cytokines in the vagina and chorioamniotic/placental membranes (Table 1).

Approximately one third of infant mortality in the US is due to pre-term delivery^61^ and infection plays a prominent role^62,63^. Taken together the obtained data suggest that polysaccharide based GBS vaccines may contribute to preventing not only infant disease but also morbidity during early stages of pregnancy.

## Methods

### Bacterial strains, growth conditions and preparation of CPSIII conjugates

GBS COH1 (type III, ATCC^®^ BAA-1176™) was grown in Todd-Hewitt broth (THB, BD) and plated on Granada medium (BD). To start liquid cultures, frozen bacteria (15% glycerol in THB) were diluted in fresh medium at an initial 600 nm absorbance (A_600_) of 0.05 and incubated statically at 37 °C without CO_2_ until exponential phase (A_600nm_ = 0.6–0.7). Bacteria were centrifuged (10 minutes at 3000 Xg and 4 °C) and suspended in fresh medium (final concentration of 1–2 × 10^9^ CFU/ml). The CPS III was extracted from GBS COH1, purified and randomly conjugated to CRM_197_ (CRM, detoxified diphtheria toxin) by reductive deamination, as previously described^64^.

### Ethical statements

All animal studies were carried out in compliance with the current Italian legislation on the care and use of animals in experimentation (Legislative Decree 26/2014) and with the GSK Vaccines Animal Welfare Policy and Standards. Protocols were approved by the Italian Ministry of Health (authorization DM292–2013B) and by the local GSK Vaccines Animal Welfare Body (authorization AWB 201307). Animals were caged in IVC conditions with food and water *ad libitum*. Groups of either four-five mice or two rats were caged together, and were separated one day before infection. Enrichments were used throughout the experimental period and removed immediately before the expected delivery date. Sterile tap water was changed every 7 days; cage and enrichment change was performed every 2 weeks. In all experiments, infected animals were monitored on a daily basis and euthanized when they exhibited defined humane endpoints that had been pre-established for the study in agreement with GSK Vaccines Animal Welfare Policies.

### *In vivo* rodent models of infection, immunization and protection experiments

The immunization and infection schedules in each of the three developed rodent models are reported in Supplementary Figure 1. GBS bacteria grown to exponential phase were intra-vaginally administered to pregnant 8/10-week-old CD1 mice (10 μl using a pipette) or pregnant 10/12-week-old Wistar-Han rats (100 μl, using a pipette).

In the mouse model of perinatal GBS infection (Supplementary Figure 1A), pregnant mice were intra-vaginally inoculated with GBS between 4 and 2 days before the expected delivery date. This two-day variability was mainly due to the two-day interval used for mating; further, pregnancy in mice and rats lasts 20–21 and 21–23 days respectively, making unsure the final delivery date. Pregnant mice were monitored twice a day until delivery and their pups were enumerated on days 1 and 2 after birth. On day 2 post-delivery, mothers and pups were sacrificed through cervical dislocation and beheading respectively, in order to collect the maternal vagina, uterus and blood, and offspring nasal washes, intestinal tract, lungs and blood. Nasal washes in sacrificed pups were performed through the pharynx after removal of lower jaw, with 200 μl of PBS using a capillary of an ART 20 μl GEL tip inserted on a 200 μl tip.

In order to investigate infection in chorioamniotic/placental membranes and in the fetus (mouse and rat ascending infection models, Supplementary Figure 1B and C respectively), animals were infected between 4 and 2 days before the expected delivery date and sacrificed 2 days later to collect maternal vaginas and chorioamniotic/placental membranes (mice and rats) and the fetal lungs (rats only). Mothers were sacrificed through cervical dislocation (mice) or CO_2_ inhalation (rats) while fetuses were beheaded. Maternal blood was collected from the cheek (mice) or from the tail vein (rats), while the offspring blood was obtained after beheading.

In all cases, the maternal vagina, uterus and chorioamniotic/placental membranes, the offspring intestinal tract and lungs and the fetal lungs where collected as whole organs and homogenized using gentleMACS™ Octo Dissociator – (Miltenyi Biotec) following supplier’s instructions; CFU counts were reported as CFU/organ. Vaginal colonization/infection was also followed across different time points by collecting vaginal swabs in place of whole organs; mouse and rat vaginal swabs were diluted in 0.2 ml PBS and 1 ml of PBS, respectively. Bacteria enumerated in vaginal and nasal washes or in swabs were expressed as CFU/wash or CFU/swab respectively. Bacterial numbers in the blood were expressed as CFU/ml. In all mouse immunization experiments, 5-weeks-old CD1 females were vaccinated intra-peritoneally on days 0, 21 and 35 with 1 μg of CPSIII-CRM adjuvanted with 2 mg/ml of Aluminum hydroxide (Alum). For the studies in rats, 6-week-old Wistar-Han females were immunized subcutaneously on days 0, 21 and 35 with the same vaccine formulation as above. In both cases females were mated on day 38 after immunization and infected 4 to 2 days before the expected delivery date as described. Bleedings for sera collection were performed 2–3 days before each immunization. Collected blood samples were plated to enumerate GBS CFU, the serum was let separate from cells at room temperature for 4–6 hours, centrifuged at 21.000 Xg for 15 minutes to remove all the cellular debris and filtered using a 0.22 μM filter. Sera were stored as previously described for ELISA, OPKA and cytokine analysis^65^.

### Measurement of anti-CPSIII IgG by Enzyme-linked immunosorbent assay (ELISA)

Microtiter plate wells (NUNC, Maxisorp) were coated with 100 ng of CPSIII conjugated to Human Serum Albumin (CPS III-HSA) via the spacer adipic acid dihydrazide in PBS pH 7.4^64^. Plates were incubated overnight at 2–8 °C, washed three times with PBST (0.05% Tween-20 in PBS pH 7.4) and saturated with 250 μL/well of PBST-B (2% Bovine Serum Albumin-BSA in PBST) for 90 min at 37 °C. Two-fold serial dilutions of test and standard sera in PBST-B were added to each well. Plates were incubated at 37 °C for 1 hour, washed with PBST, and then incubated for 90 min at 37 °C with anti-mouse IgG-alkaline phosphatase (Sigma) diluted 1:2000 or anti-rabbit IgG-alkaline phosphatase diluted 1:1000 in PBST-B. After washing, the plates were developed with a 4 mg/mL solution of p-Nitrophenyl Phosphate (pNPP) in 1 M diethanolamine (DEA) pH 9.8, at room temperature for 30 min. After blocking with 7% w/v EDTA, the absorbance was measured using a SPECTRAmax plate reader with wavelength set at 405 nm. IgG concentrations were expressed as relative ELISA Units/mL (EU/mL) and calculated by interpolating the absorbance values of serial sample dilutions on the standard calibration curve (Reference line method). The Standard consisted of a pool of hyperimmune sera obtained from animals immunized with 3 doses of CPSIII-CRM adjuvanted with Alum.

### GBS opsonophagocytic killing assay

Opsonophagocytic killing assays (OPKA) were conducted on pooled sera from immunized mice as previously described^66^. Briefly, the reaction was performed in 96 well plates (Nunc), in HBSS (Hank’s Balanced Salt Solution, Gibco). For each reaction mixture, heat inactivated (56 °C for 30 minutes; HI) test serum, GBS COH1 bacteria, differentiated HL-60 cells and 10% baby rabbit complement (Cedarlane) were added. Control reactions were performed in the presence of HI complement or in the absence of antibodies or effector cells. For each serum sample, four dilutions were tested. Bacteria were prepared by directly diluting frozen aliquot stocks (A_600_ 0.45–0.5) in HBSS + 10% normal rabbit serum (Sigma) to obtain a final concentration of 6 × 10^4^ CFU/well (effector cells to GBS ratio 25:1). The reaction was incubated 1 hour at 37 °C with shaking (300 rpm). At times 0 and 60 minutes (T0 and T60) reactions were diluted in sterile water and 10 μL were plated in trypticase soy agar containing 5% blood sheep (Particle Measuring Systems). Plates were incubated overnight at 37 °C in presence of 5% CO_2_ and the percent of killing was calculated as (mean CFU at T0 - mean CFU at T60)/(mean CFU at T0). OPKA titers were expressed as the reciprocal serum dilution mediating 50% bacterial killing, estimated through linear interpolation of the dilution-killing OPKA data. The Lower Limit of Detection of the assay was 1:30.

### Cytokine analysis

Cytokine analyses in the blood and tissue homogenates of vaccinated and control rats were performed using the Bio-Plex Pro Rat cytokine 24-plex panel (Bio-rad) following manufacturer’s procedures. Reactions were read at the Luminex^®^ 200 system. The complete list of analyzed cytokines includes EPO, G-CSF, GM-CSF, GRO/KC (IL-8), IFN-γ, IL-1α, IL-1β, IL-2, IL-4, IL-5, IL-6, IL-7, IL-10, IL-12p70, IL-13, IL-17, IL-18, M-CSF, MCP-1, MIP-1α, MIP-3α, RANTES, TNF-α, and VEGF. Only cytokines showing a statistically significant difference between vaccinated rats and the control group were reported.

### Tissue processing, histopathological evaluation, and immunohistochemistry

Tissues were fixed for 18–24 h in 4% buffered formaldehyde (Carlo Erba), washed and embedded in paraffin (formaldehyde-fixed and paraffin embedded, FFPE) with standard techniques. Then, 5 µm-thick sections were cut and stained with hematoxylin and eosin (H&E). Histopathological examination was performed in blind by experienced veterinary pathologists. Tissues were examined to detect signs of inflammation, and in particular the presence of mononuclear and polymorphonuclear (PMN) cells, interstitial diffuse inflammation, interstitial foci and glandular damage. Adjacent sections were subjected to immunohistochemical staining (IHC) for the detection of GBS in the automated immunostainer Discovery XT (Ventana). Briefly, sections were de-paraffinized, subjected to heat-mediated antigen retrieval in basic buffer (CC1, Ventana), saturated with casein-based solution (Antibody block, Ventana) and incubated for 12 h at room temperature in presence of anti-GBS serum from rabbits immunized with CPSIII-CRM/Alum diluted 1:400. Sections were further incubated with alkaline phosphatase-conjugated anti-rabbit antibody (UltraMap anti-Rb AP, Ventana) for 16 min at 37 °C, and finally with a chromogenic solution (ChromoMapBlue, Ventana) for 28 min at 37 °C. Counterstaining was performed with Red Counterstainer 2 (Ventana). Neutrophils were detected with anti-myeloperoxidase (MPO) antibody (antibody from Abcam ab9535, raised in rabbits, at the final concentration of 10 µg per slide) incubated for 12 h at room temperature, followed by HRP-conjugated anti-rabbit antibody (OmniMap anti-Rb HRP, Ventana) for 24 min at 37 °C, and finally by the chromogenic solution (ChromoMapDAB, Ventana) for 28 min at 37 °C. Counterstaining was performed with Hematoxylin II (Ventana). Slides were examined and images acquired by an optical microscope (Leica DM 5500B).

### Statistics

Statistical analyses were performed using the Graph Pad Prism^®^ 7 software. The Mann-Whitney U-test (two tailed), the Fisher’s exact test (two tailed) or the Wilcoxon signed rank sum Test were used as reported to calculate statistical significance. P values of < 0.05 were considered statistically significant. Legend: *p-val < 0.05; **p-val < 0.01; ***p-val < 0.001; ****p-val < 0.0001.

### Data availability

The datasets generated during and/or analyzed during the current study are available from the corresponding author on reasonable request.

## Electronic supplementary material

Supplementary figure 1
