# Supplementary material for: Protective effect of Group B Streptococcus type-III polysaccharide conjugates against maternal colonization, ascending infection and neonatal transmission in rodent models
Source: Sci Rep. 2018 Feb 7;8:2593. doi: 10.1038/s41598-018-20609-5 (PMC5803199; doi:10.1038/s41598-018-20609-5)
Supplement: Supplementary file 1 — Supplementary figure 1 [file 41598_2018_20609_MOESM1_ESM.doc]

**Protective effect of Group B Streptococcus type-III polysaccharide conjugates against maternal colonization, ascending infection and neonatal transmission in rodent models**

Emiliano Chiarot^a,$^, Angela Spagnuolo^a,$^, Silvia Maccari^a^, Eleonora Naimo^a,b^, Alessandra Acquaviva^a^, Raffaella Cecchi^a^, Bruno Galletti^a^, Monica Fabbrini^a^, Elena Mori^a^, Paolo Ruggiero^a^, Guido Grandi^a,c^, Maria Rita Fontana^a^, Giuliano Bensi^a^* and Immaculada Margarit^a^*

**Affiliation:**

^a^GSK, Siena, Italy

**Present address:**

^b^Hannover Medical School, institute of virology, Hannover, Germany

^c^University of Trento, Centro di Biologia Integrata – CIBIO, Trento, Italy

^$^ = These authors contributed equally and are listed in alphabetical order

* = Corresponding author

**Corresponding authors**

Immaculada Margarit Y Ros, GSK Vaccines Srl, Via Fiorentina 1, Siena, Italy 53100. Telephone: +39 0577 243809; Fax: +39 0577 243564

Email: immaculada.x.margarit-y-ros@gsk.com

Giuliano Bensi, GSK Vaccines Srl, Via Fiorentina 1, Siena, Italy 53100. Telephone: +39 0577 245231; Fax: +39 0577 243564

Email: [giuliano.x.bensi@gsk.com](mailto:giuliano.x.bensi@gsk.com)

**Supplementary information**

**Supplementary figure S1**


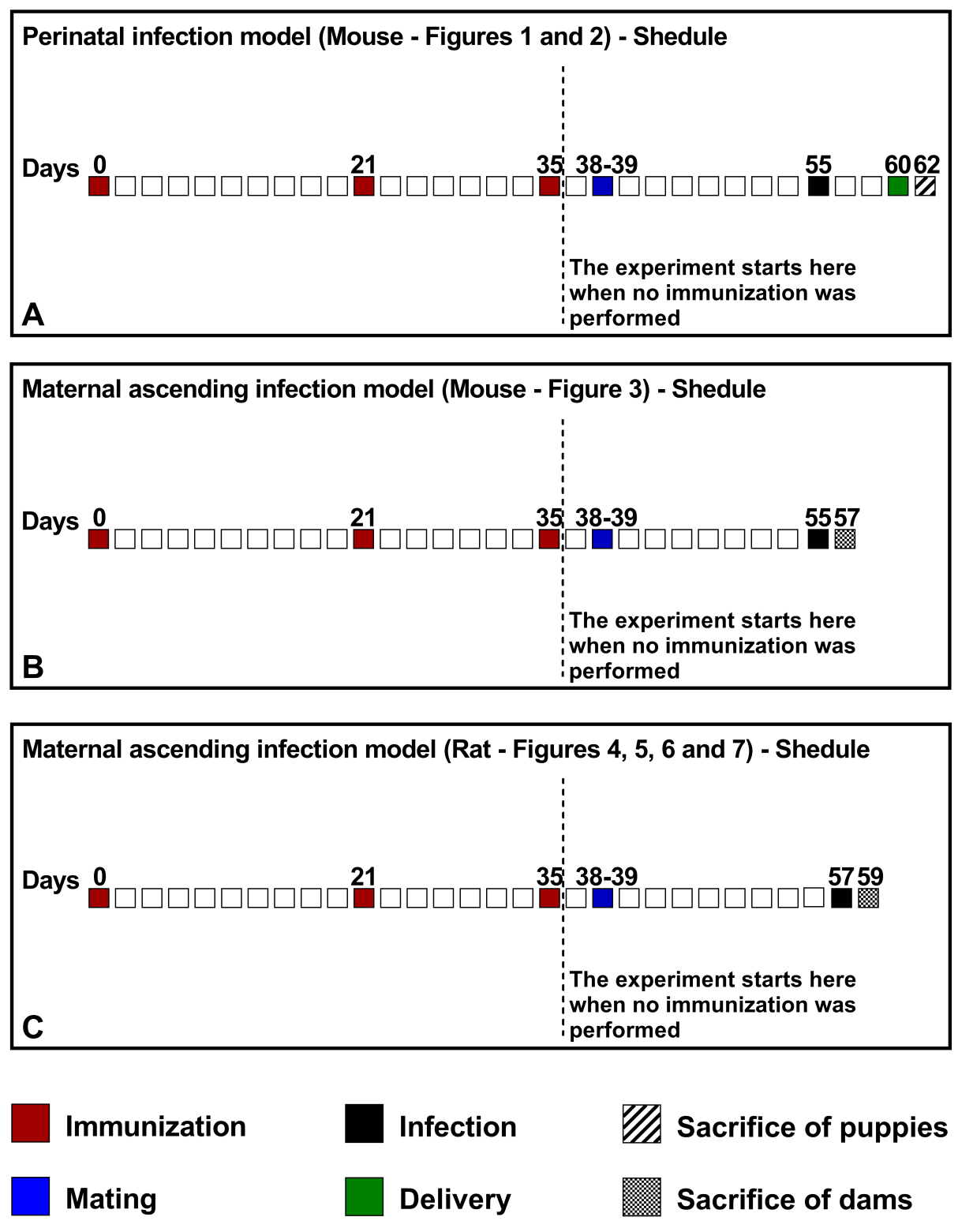


**Supplementary figure S1. Immunization and infection schedules of murine and rat models described in the manuscript.**
